# Predicting Agents That Can Overcome 5-FU Resistance in Colorectal Cancers via Pharmacogenomic Analysis

**DOI:** 10.3390/biomedicines9080882

**Published:** 2021-07-24

**Authors:** Tsui-Chin Huang, Kuan-Chieh Peng, Tzu-Ting Kuo, Li-Chun Lin, Bai-Chia Liu, Shu-Ping Ye, Chien-Chou Chu, Shih-Min Hsia, Hsin-Yi Chang

**Affiliations:** 1 Graduate Institute of Cancer Biology and Drug Discovery, College of Medical Science and Technology, Taipei Medical University, Taipei 11031, Taiwan; tsuichin@tmu.edu.tw (T.-C.H.); pkj@tmu.edu.tw (K.-C.P.); sabrina.yeh1603@gmail.com (S.-P.Y.); chienchou02@hotmail.com (C.-C.C.); 2 PhD Program for Cancer Molecular Biology and Drug Discovery, College of Medical Science and Technology, Taipei Medical University and Academia Sinica, Taipei 11031, Taiwan; d621107003@tmu.edu.tw (T.-T.K.); d621105001@tmu.edu.tw (L.-C.L.); 3 Master Program in Clinical Pharmacogenomics and Pharmacoproteomics, College of Pharmacy, Taipei Medical University, Taipei 11031, Taiwan; 4 TMU Research Center of Cancer Translational Medicine, Taipei Medical University, Taipei 11031, Taiwan; 5 Cancer Center, Wan Fang Hospital, Taipei Medical University, Taipei 11031, Taiwan; 6 Graduate Institute of Metabolism and Obesity Sciences, College of Nutrition, Taipei Medical University, Taipei 11031, Taiwan; deamily@tmu.edu.tw (B.-C.L.); bryanhsia@tmu.edu.tw (S.-M.H.); 7 School of Nutrition and Health Sciences, College of Nutrition, Taipei Medical University, Taipei 11031, Taiwan; 8 School of Food and Safety, Taipei Medical University, Taipei 11031, Taiwan; 9 Nutrition Research Center, Taipei Medical University Hospital, Taipei 11031, Taiwan; 10 Taipei Medical University and Affiliated Hospitals Pancreatic Cancer Groups, Taipei Cancer Center, Taipei Medical University, Taipei 11031, Taiwan

**Keywords:** 5-FU resistance, colorectal cancer, drug repurposing, Genomics of Drug Sensitivity in Cancer, Connectivity Map

## Abstract

5-Fluorouracil (5-FU) is one of several chemotherapeutic agents in clinical use as a standard of care to treat colorectal cancers (CRCs). As an antimetabolite, 5-FU inhibits thymidylate synthase to disrupt the synthesis and repair of DNA and RNA. However, only a small proportion of patients benefit from 5-FU treatment due to the development of drug resistance. This study applied pharmacogenomic analysis using two public resources, the Genomics of Drug Sensitivity in Cancer (GDSC) and the Connectivity Map, to predict agents overcoming 5-FU resistance in CRC cells based on their genetic background or gene expression profile. Based on the genetic status of adenomatous polyposis coli (APC), the most frequent mutated gene found in CRC, we found that combining a MEK inhibitor with 5-FU exhibited synergism effects on CRC cells with APC truncations. While considering the gene expression in 5-FU resistant cells, we demonstrated that targeting ROCK is a potential avenue to restore 5-FU response to resistant cells with wild-type APC background. Our results reveal MEK signaling plays a pivotal role in loss-of-function, APC-mediated 5-FU resistance, and ROCK activation serves as a signature in APC-independent 5-FU resistance. Through the use of these available database resources, we highlight possible approaches to predict potential drugs for combinatorial therapy for patients developing resistance to 5-FU treatment.

## 1. Introduction

Colorectal cancer (CRC) is the second leading cause of cancer death, with an estimated 147,950 new CRC cases and 53,200 deaths in the United States in 2020 [1]. Administration of 5-fluorouracil (5-FU) has been one of the standard chemotherapy regimens both alone and in combination with other agents for colon cancers for almost five decades. 5-FU is an antimetabolite that inhibits thymidylate synthase to disrupt synthesis and repair of DNA and RNA, resulting in cell death. However, 5-FU has been found to be only 20% effective, and the response rate of advanced CRC patients treated with 5-FU/leucovorin is about 10–15% [2,3]. A notable proportion of the patients show tumor recurrence after 5-FU treatment that is mainly caused by drug-resistant cancer cells.

The most common mutations in colon cancer are found in adenomatous polyposis coli (APC) and resulted in inactivated truncation forms. APC controls the activity of β-catenin, particularly in the Wingless/WNT signaling pathway. APC protein typically builds a complex with glycogen synthase kinase 3β (GSK-3β) and AXIN through physical interactions with the 20 AA and SAMP repeats. This complex is then able to bind β-catenin in the cytoplasm, where β-catenin dissociates from adherent contacts between cells. β-catenin is subsequently phosphorylated by GSK-3β followed by casein kinase (CK1)-triggered initial phosphorylation. Phosphorylated β-catenin is then targeted for ubiquitination and degraded by the cellular proteasome. This prevents it from translocating into the nucleus, where it acts as a transcription factor and forms a complex with TCF4, promoting transcription of the proto-oncogene and cell cycle regulator c-MYC, the G1/S-regulating Cyclin D, the gene encoding the matrix-degrading metalloproteinase, matrilysin, the AP-1 transcription factors c-JUN and FRA1, and the urokinase-type plasminogen activator receptor genes [4,5,6,7]. In addition to regulating β-catenin, APC is responsible for various cellular processes, including cytoskeletal integrity, cellular adhesion, and DNA repair [8,9,10,11]. The versatile role of APC suggests its mutations would contribute to a wide range of controlling or modulating cellular physiology, including 5-FU resistance in CRC. Recent studies revealed that APC mutations play a critical role in colorectal cancer patients acquiring resistance to clinical 5-fluorouracil treatment, indicating a limited CRC treatment outcome remains to be improved [12].

Several studies have applied transcriptome profiling techniques such as RNA-seq and microarray on mRNA and miRNA expression levels in CRC cells established with 5-FU resistance [13,14,15]. Furthermore, valuable resources of systematic assessments on drug responses and multiplexed genomic manipulation data across model cancer cell lines are now distributed to the cancer research community, facilitating our understanding of drug resistance in CRC. To predict potential drugs to overcome 5-FU resistance, we performed the analysis in a genetic background-dependent manner.

We utilized the APC status-related drug responses from the Genomics of Drug Sensitivity in Cancer (GDSC) [16]. We validated the proposed drug, MEK inhibitors, to overcome the APC dysfunction-dependent 5-FU resistance in six CRC cell lines. To correlate our finding to the clinical evidence, we compared the phosphoprotein levels obtained from The Cancer Genome Atlas (TCGA) database [17] with the APC truncation status in cBioPortal [18,19]. We confirmed that CRC patients carrying truncated APC exhibited higher phosphorylations in MAPK1 and MAPK2 than patients bearing full-length APC.

For the APC-independent 5-FU resistance, we applied a cellular context-based prediction on HCT116 cells, which express wild-type APC. We analyzed the gene expression profiles acquired from 5-FU resistant HCT116 cells and their parental line to identify the differential gene expression. By reversely matching the 5-FU gene signatures to drug-induced patterns in the Connectivity Map database [20], we found that the RHO kinase inhibitor can sensitize resistant HCT116 cells to 5-FU treatment.

## 2. Materials and Methods

### 2.1. Retrieval of the Effect of APC Mutation on Drug IC50s from Genomics of Drug Sensitivity in Cancer (GDSC)

The effect of APC mutation on IC50 values in response to drugs in Pan-Cancer analysis from GDSC1 and GDSC2 and the drug annotation were downloaded from two screenings, GDSC web portal (https://www.cancerrxgene.org/ (accessed on 21 July 2019). The positive values of the effect of APC mutation on drug sensitivity (represented as IC50 effect) indicate drug resistance, and the negative values show drug sensitivity. We selected the drug responses that were commonly tested in both screenings and ranked the drug by the average calculated effect size of each category. The distribution of effects was categorized by the mechanism of action of drugs based on the target pathway.

### 2.2. Cell Culture and Cell Viability Measurement

Human colorectal carcinoma cells HCT116, HT29, DLD1, and SW620 were obtained from the American Type Culture Collection (ATCC, Manassas, VA, USA). All cells were maintained in RPMI 1640 (Gibco Laboratories, Grand Island, NY, USA) supplemented with 10% fetal bovine serum (Gibco Laboratories) at 37 °C in a humidified incubator with 5% CO_2_. To establish a 5-FU-resistant HCT116 subline, we repeatedly exposed expose HCT116 cells to stepwise increasing concentrations of 5-FU over a period of ~12 months to obtain 5-FU-resistant HCT116 (HCT116-5FUR) with IC_50_ over 50 μM. All cells were verified as mycoplasma-free by a PCR-based detection.

### 2.3. Constructs and Transfection

pCMV-Neo-Bam APC (Addgene plasmid #16507), pCMV-Neo-Bam APC 1-1309 (Addgene plasmid #16509), pCMV-Neo-Bam APC 1-1941 (Addgene plasmid #16510), pCMV-Neo-Bam APC 1-2644 (Addgene plasmid #16511) were gifts from Bert Vogelstein. HCT116 cells were seeded into 6-well plates and transfected with 2 μg of vector pCMV harboring various truncated APCs, from 1-1309, 1-1941, 1-2644, full-length APC, or an empty one using lipofectamine 3000 (Invitrogen, Grand Island, NY, USA) for 48 h before drug treatment.

### 2.4. MTS Assay

A total of 2500 cells were seeded into a 96-well plate for 16 h and treated with 5-FU (Sigma-Aldrich, St. Louis, MO, USA), PD-0325901 (Cayman Chemical, Ann Arbor, MI, USA), 3-(4-Pyridyl)indole (RHO-kinase-inhibitor-III, Rockout) (Cayman Chemical), and BMS-754807 (Cayman Chemical) at indicated concentrations from 1000 × stocks to a final concentration of DMSO as 0.1%. Cell viability was measured by MTS assay using the CellTiter 96^®^ AQueous One Solution Cell Proliferation Assay kit (Promega, Madison, WI, USA) according to the manufacturer’s instruction. The absorbance was detected at 490 nm with an Epoch Microplate Spectrophotometer (BioTek Instruments, Winooski, VT, USA).

### 2.5. Gene Expression Dataset and Data Analysis

The expression dataset of HCT116 parental and HCT116 5-FU resistant cells (E-MEXP-390) [21] was obtained from the Array Express database (http://www.ebi.ac.uk/arrayexpress/ (accessed on 7 October 2019)). The Affymetrix CEL file data was processed by the RMA (Robust Multichip Average) method, including background correction, quantile normalization, and log2 transformation, and the differentially expressed genes were defined using limma package [22]. Overexpressed genes were inverted and searched against the perturbation-induced gene expression profiles in the Connectivity Maps 2.0.

### 2.6. Functional Enrichment Analysis

Differentially expressed genes were submitted to the Database for Annotation, Visualization, and Integrated Discovery (DAVID) v6.8 (https://david.ncifcrf.gov/home.jsp (accessed on 12 November, 2019)) [23] for functional enrichment analysis using Gene Ontology terms, according to their molecular function (MF), biological process (BP), and cellular component (CC). Human Genome U133 Plus 2 Array was selected as backgrounds. The *p*-value was corrected for multiple testing by Bonferroni correction.

### 2.7. Western Blot Analysis

PBS washed cells were harvested and lysed in RIPA buffer (20 mM Tris-HCl (pH 7.5), 150 mM NaCl, 1 mM EDTA, 1 mM EGTA, 1% NP-40, and 1% sodium deoxycholate) containing the Halt Protease and Phosphatase Inhibitor Cocktail (Thermo Fisher, Waltham, MA, USA). Protein concentration was determined using the T-Pro BCA Protein Assay Kit (T-Pro biotechnology, Taipei, Taiwan). Protein samples were separated by SDS-PAGE, transferred onto PVDF membranes (Millipore, Billerica, MA, USA), and blocked with Block PRO blocking buffer (Visual Protein Biotechnology Corporation, Taipei, Taiwan). Blot was incubated with a primary antibody followed by incubation of horseradish peroxidase-conjugated secondary antibodies. Protein level was determined by enhanced chemiluminescence (Advansta, Menlo Park, CA, USA) detection. Antibodies probing p-AKT (S473), AKT, p-ERK1/2 (Y202/Y204), ERK1/2, and GAPDH (Cell Signaling Technology, Inc., Danvers, MA, USA) were used.

### 2.8. Clinical Data Analysis

Clinical data of CRC patients investigated by The Cancer Genome Atlas (TCGA) program were obtained from cBioPortal (http://www.cbioportal.org/index.do (accessed on 20 April 2018) [18,19]. Data of protein expression and phosphorylation levels were retrieved from antibody-based reverse-phase protein array (RPPA) profiling of CRC patients. The differences between patients with truncated APC and with wild-type APC were compared using Student’s *t*-test followed by the Benjamini–Hochberg procedure to control the false discovery rate.

### 2.9. Statistical Analysis

All experiments were performed at least three times independently. Data were expressed as mean ± SD. Unpaired two-tailed t-tests were used for the comparison of two groups, and *p*-values < 0.05 were considered significant. To calculate the drug combination effect, the Combination Index (CI) was assessed using CompuSyn software version 1.0 (Combosyn, Inc, Paramus, NJ, USA) [24]. A CI greater, lesser, or similar than 1 indicates antagonism, synergism, or additive effect, respectively.

## 3. Results

### 3.1. Cell Overexpressing 1-1309 Truncated APC Acquires 5-FU Drug Resistance

**Figure 1:**
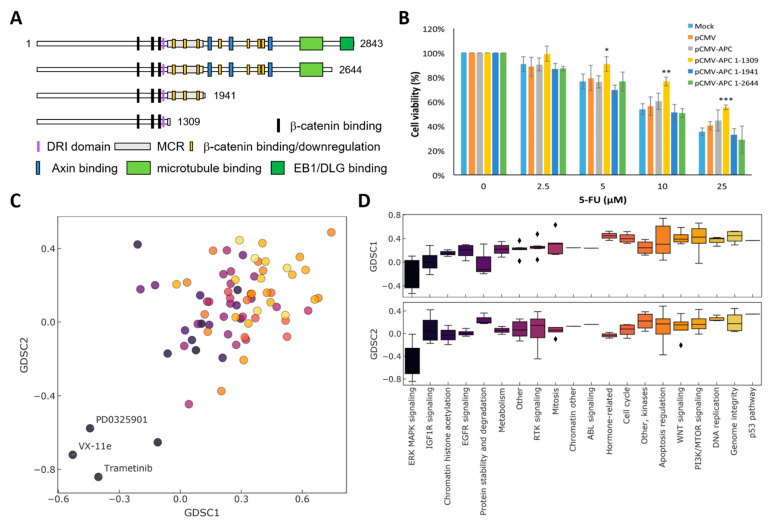
APC status and drug response: (**A**) C-terminal protein domains of wild-type (1-2843) and truncated APCs. Domains of APC are subsequently deleted as indicated: EB1/DLG-binding domains (1-2644), AXIN-binding (1-1941), and mutation cluster region (MRC) and β-Catenin binding/downregulation sites (1-1309). (**B**) HCT116 cells were untransfected (Mock) or transfected with pCMV harboring various truncated APCs illustrated in (**A**) with lipofectamine 3000 for 48 h. The expressing length of APC is indicated. pCMV-APC represents the full-length APC, and pCMV indicates the empty vector. After transfection, 2500 cells were seeded into 96-well plates for 16 h and treated with 5-FU at the concentration of 2.5, 5, 10, and 25 μM for 48 h. Cell viability was then accessed by MTS assay and normalized to DMSO vehicle control. *, *p* < 0.05; **, *p* < 0.01; ***, *p* < 0.001. (**C**) APC status correlated drug sensitivity prediction obtained from Genomics of Drug Sensitivity in Cancer (GDSC) database. The APC mutation effects on drug sensitivity were plotted from two independent screening, GDSC1 and GDSC2. Each drug is colored by its targeting pathway in terms of the mechanism of action. Top drugs targeting ERK MAPK signaling contributed to increased sensitivity are indicated. (**D**) The effect of APC mutation on drug response is categorized by the target pathway and ranked by the mean of effects from GDSC1 and GDSC2. The color code is the same as (**C**). A negative effect indicates improved sensitivity.

To examine whether APC truncation participates in 5-FU resistance in CRC, we transfected APC mutants (Figure 1A) lacking EB1/DLG-binding (APC 1-2644), microtubule- and AXIN-binding (APC 1-1941), and β-Catenin binding and downregulation (APC 1-1309) domains, respectively, into HCT-116 cells that carry full-length APC. We measured the cell viability by MTS assay after 48 h of 5-FU treatment and compared that of cells transfected with truncated APCs to untransfected cells, cells transfected with empty vector (pCMV), or cells overexpressing wild-type APC. We found that only the 1-1309 truncated APC lacking β-catenin binding/downregulation sites significantly exhibited tolerance to 5-FU treatment (Figure 1B), suggesting that loss of binding of β-catenin or disrupting the local structure of nearby DRI domain is crucial for 5-FU sensitivity.

### 3.2. APC Mutations and Drug Sensitivity

**Figure 2:**
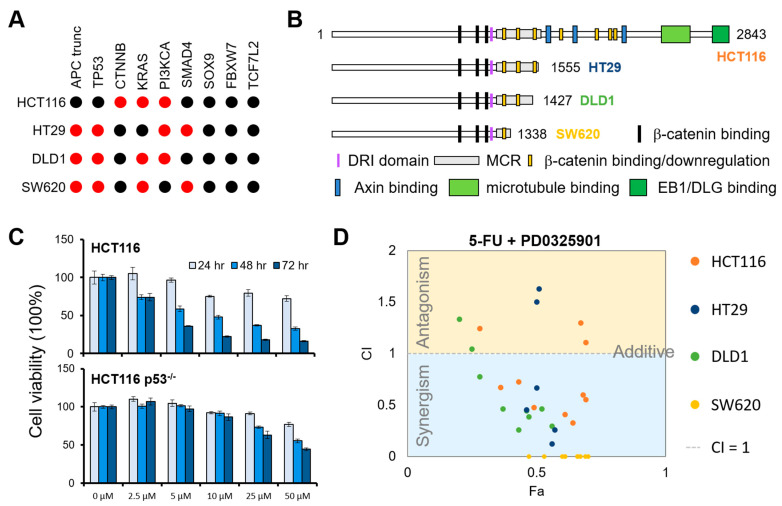
Combinatorial effects of 5-FU with targeting p53 pathway or ERK/MAKP signaling: (**A**) The genetic status of frequently mutated genes in CRC cell lines was used. Wild-type and genes harboring mutations are colored in black and red, respectively. (**B**) APC status in cell lines was tested. Cell lines were colored corresponding to the data in (**D**). (**C**) Inhibition of p53-mediated pathway contributed to 5-FU resistance. P53 null HCT116 (p53^−/−^) cells or parental HCT116 cells were treated with 5-FU for 24, 48, and 72 h at indicated concentrations. Cell viability was measured by MTS assay and normalized to DMSO treated control. (D) Quantitation of synergism and antagonism in MEK inhibitor and 5-FU combination. Fraction affected (Fa) versus Combination Index (CI) plots were generated by CompuSyn. CI indicates a greater/synergism (CI < 1), lesser/antagonism (CI > 1), or similar (CI = 1) effect than the expected additive effect.

To understand whether and to what extent the APC mutation correlates with drug sensitivity, we extracted the data from GDSC [16] to survey the correlation between APC mutation and drug sensitivity from the IC50 values of 448 anti-cancer drugs to 851 and 720 cancer cell lines from GDSC1 and GDSC2, respectively (Figure 1C). The differences between mean IC50 from cells with APC mutants and cells with wild-type APC were used to present the effect of APC mutation on drug sensitivity. The negative value indicates increased drug sensitivity of a given drug to cells with APC mutation. To gain the global response of anti-cancer drugs to APC mutation, we classify the effects according to the drug targeting pathway (Figure 1D). Compounds targeting the p53 pathway, genome integrity, DNA replication, and PI3K/mTOR signaling are the most frequent unresponsive drugs to APC-mutated cells, echoing the lower sensitivity to 5-FU treatment in cells carrying the 1-1309 APC mutant (Figure 1B) and supporting 5-FU sensitivity in p53-dependent cells with wild-type APC (Figure 2C). On the other hand, cells with APC mutations are sensitive to drugs targeting ERK/MAPK signaling, mainly MEK inhibitors, intriguing us to combine these drugs to increase the drug response to 5-FU.

### 3.3. MEK Inhibitor PD-0325901 Sensitizes APC Mutation Containing CRC Cells to 5-FU Treatment

To validate whether targeting the p53 pathway causes drug resistance in cells with wild-type APC, we treated p53 null HCT116 cells and their parental cells with 5-FU in a dose- and time-dependent manner. We found that inhibiting p53 signaling under an APC wild-type background enhanced 5-FU resistance, confirming the synergic role of p53 in APC mutation (Figure 2C). Next, we validate whether ERK/MAPK singling inhibitors can overcome APC mutation caused by 5-FU resistance. We selected one of the MEK inhibitors, PD-0325901, and examined its cytotoxicity in four colon cancer cell lines. Each cell line has different truncated APCs (Figure 2B) except for HCT116 cells, which express full-length APC but harbor gain-of-function mutations in β-Catenin (Figure 2A). We treated the cells with PD-0325901 alone or combined with 5-FU in dose-dependent manners. As expected, SW620 cells harboring the shortest truncated APC showed the highest survival rate under 5-FU treatment, suggesting 5-FU-inhibited cell proliferation is correlated with APC-interacting downstream events (Figure 2D and Figure S1). To testify the hypothesized roles of MEK activity to compensate for 5-FU sensitivity, we treated these CRC cells with PD-0325901 in combination with 5-FU. To examine the drug effects, we applied the Combination Index (CI), a standard measure of combination effect. A CI indicates a greater (CI < 1), lesser (CI > 1), or similar (CI = 1) effect than the expected single additive effect expected from the knowledge of the effects of each drug individually. We found that the MEK inhibitor PD-0325901 displayed cell line-dependent effects with a strong synergic effect (CI < 0.1) in SW620 cells, a moderate synergic effect in DLD1 and HCT116 cells, and an antagonistic effect in HT29 cells (Figure 2D), suggesting the activation of MEK1/2 might be involved in but not required for developing 5-FU resistance.

### 3.4. ERK1/2 Phosphorylation Is Induced in Patients with Truncated APC

**Figure 3:**
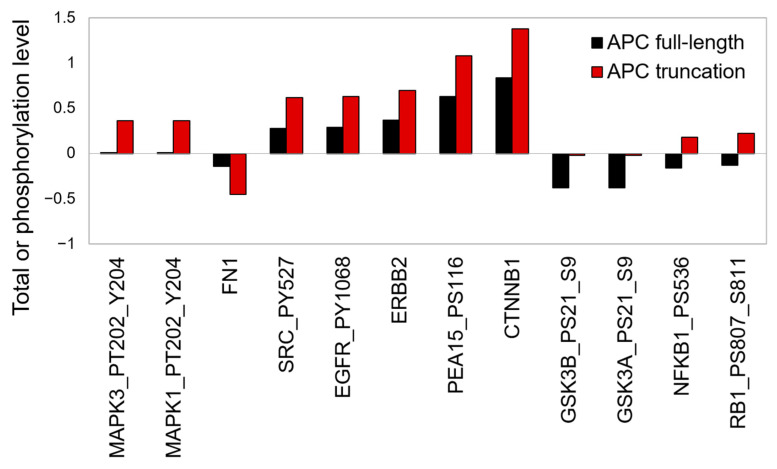
ERK1/2 phosphorylation in the TCGA cohort is associated with APC truncation. The protein expression and phosphorylation levels of CRC patients (COAD in TCGA) were retrieved and compared between APC full-length (black bars) and truncated APC (red bars) patients. Data with a q-value (adjusted *p*-value by Benjamini–Hochberg correction) less than 0.05 are shown and ranked based on fold changes. Phosphorylation information is annotated by official gene symbol followed by phosphorylation sites.

To explore what molecular clues were in line with APC truncation clinically, we compared the protein expression and phosphorylation from CRC patients baring wild-type or truncated APC from TCGA. We found that pERK1/2 (pT202/Y204 on MAPK1 and MAPK3) were the most upregulated proteins with 36-fold induction in patients carrying truncated APC compared with patients baring wild-type APC (Figure 3, q-value = 6.13 × 10^−7^), demonstrating that APC truncation is associated with ERK1/2. We suggest that APC status might serve as a molecular signature for CRC patients to exhibit a positive response from the MEK inhibitor in combination with 5-FU.

### 3.5. Function of Extracellular Ligand Binding to Regulate Cell Mobility Was Upregulated in 5-FU Resistant Cells

**Figure 4:**
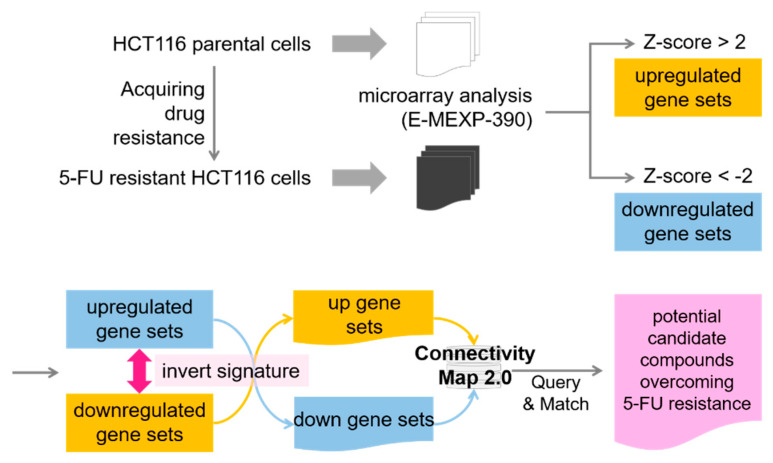
Pattern matching analysis to reveal drugs able to overcome APC-independent 5-FU resistance. The differentially expressed genes from 5-FU-resistant and parental HCT116 cells were defined and inverted to match the drug-responsive gene expression pattern in the Connectivity Map 2.0 database. The drugs triggering negatively correlated gene expression patterns are reported as potential candidate compounds overcoming 5-FU resistance.

Next, we sought to discover the molecular signatures in APC-dependent 5-FU resistance for combinatorial therapy. Consequently, we analyzed the gene expression profiles of APC-wild-type HCT-116 cells and the derived 5-FU resistant subline (Figure 4). There were 869 genes differentially expressed, with 456 upregulated and 413 downregulated. The differentially expressed genes were then subjected to functional enrichment analysis by DAVID. The selectively enriched functions of differentially expressed genes are listed in Tables S1 and S2, respectively. We found that the downregulated genes in 5-FU-resistant cells were involved in RNA processing and metabolism as well as folic acid metabolism (Table S2), suggesting that 5-FU resistance might be caused by abrogating the nucleoside biosynthesis pathway, which 5-FU interrupts. On the other hand, the top enriched terms in upregulated genes are receptor binding, cell migration, and extracellular space from receptor binding in MF, BP, and CC, respectively (Table S1).

### 3.6. Reversed Expression Predicts ROCK Activation in 5-FU Resistant Cells

**Figure 5:**
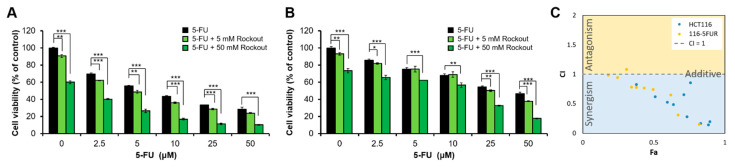
ROCK inhibitor synergizes 5-FU effects on resistant cells: (**A**) HCT116 and (**B**) 116-5FUR cells were treated with Rockout and 5-FU at the indicated doses. We measured the cell viability by MTS assay and normalized it to DMSO vehicle control. Grass green and green bars are cell viability of ROCK inhibitor Rockout-treated cells at different dosages compared with 5-FU alone treated cell viability. *, *p* < 0.05; **, *p* < 0.01; ***, *p* < 0.001. (**C**) Quantitation of synergism and antagonism in Rockout inhibitor and 5-FU combination. Fa versus CI plots were generated by CompuSyn. CI indicates a greater/synergism (CI < 1), lesser/antagonism (CI > 1), or similar (CI = 1) effect than the expected additive effect.

To discover possible inhibitors to restore 5-FU drug sensitivity in cells with wild-type APC, we inversed the differential gene regulation of 5-FU-resistant cells to parental ones. Subsequently, we submitted the list to query the Connectivity Map database (Figure 5). The query results generated a list of potent candidate drugs whose effects matched the expression signature inverted to 5-FU. We listed the top 10 results of pharmacological perturbations in Table 1. The inhibition on ROCK demonstrated the highest correlation in inverse gene expression of 5-FU resistant cells, showing the participation of the ROCK pathway in 5-FU resistance establishment. As the major downstream effector of small GTPase RhoA, ROCK is involved in the regulation of actomyosin cytoskeleton to promote contractile force generation for cell mobility [25,26]. This feature was in agreement with our previous findings that metastatic activity is gained in 5-FU resistant cells through receptor binding from extracellular stimulation, which promotes cell migration (Table S1).

### 3.7. ROCK and IGF1R Inhibitions Overcome Resistance to 5-FU Treatment

To confirm the result of predicted drugs overcoming acquired 5-FU resistance, we independently established a 5-FU-resistant HCT116 cell line (116-5FUR). The IC_50_ at 72 h of 116-5FUR and parental cell line is 2.15 μM and 22.52 μM, respectively. We treated the 116-5FUR and parental cells with 5-FU in combination with ROCK inhibitor Rockout. Rockout presented efficient cytotoxicity in both HCT116 and 116-5FUR cells (Figure 5). As predicted, 116-5FUR cells responded to Rockout alone. Moreover, they were sensitive to 5-FU in the presence of Rockout (Figure 5B). We showed that 5 μM of Rockout sensitized the 116-5FUR cells to 5-FU at an IC50 of 14.8 μM, more than a three-fold reduction compared with the IC50 of 5-FU in 116-5FUR cells (50 μM). The synergistic effect of Rockout and 5-FU was observed in 116-5FUR and parental cells (Figure 5C), suggesting the RhoA/ROCK axis was activated in 116-5FUR cells to promote their migratory ability through extracellular signaling. IGF1R activation can trigger ROCK indirectly by forming a complex with leukemia-associated RhoGEF-12 (LARG), which in turn activates RhoA [27]. We also observed that IGF1R downstream signaling, including AKT and ERK1/2 phosphorylation, were activated in 116-5FUR cells and even higher while treating with 5-FU (Figure S2A), implying IGF1R activation might be involved in 5-FU resistance. To investigate whether blockade of IGF1R signaling improved the 5-FU response in 116-5FUR cells, we treated the cells with a combination of the 5-FU and IGF1R inhibitor BMS-754807, revealing that 1 μM of BMS-754807 sensitized 116-5FUR cells to 5-FU at an IC50 of 6.53 μM, which is comparable with that of parental cells with a 5-FU IC50 (Figure S2B).

## 4. Discussion

Overactivation of WNT signaling through β-catenin is predominantly involved in the development of CRC. About 80% of CRC patients carrying loss-of-function mutations in APC and about 5% carrying activating mutations in β-catenin are reported in The Cancer Genome Atlas (TCGA) project [28]. Most APC mutations result in C-termini truncated forms, suggesting that the protein conformation and protein–protein interactions of APC are critical to maintaining its normal function. For instance, the DNA repair inhibitory (DRI) domain of APC interacts with two DNA repair molecules, DNA polymerase β (POLβ) and flap endonuclease 1 (FEN1), to block the POLβ-directed base excision repair (BER) pathway and lead to apoptosis [11]. Here, we reported that overexpression of truncated APC 1-1309 (Figure 1) and cell lines with shorter APC forms (Figure 2) exhibited higher tolerance to 5-FU treatment. The result suggests that the DRI domain and downregulation of the β-catenin region in APC contribute to 5-FU sensitivity.

Deming et al. [29] indicated that mutation in PIK3CA simultaneously occurred with APC mutation in CRC, highlighting the resistance on PI3K inhibitors in APC-mutated cells. GDSC analysis also indicates that except for p53-mediated DNA repair and genome instability, PI3K/mTOR signaling ranked as the fourth resistant pathway related to APC mutation (Figure 1D). We postulated this might be due to the co-occurrence of the PI3K mutation with mutated APC. On the other hand, we showed that the MEK inhibitor exhibited a positive response in APC-mutated and β-catenin-activated cells (Figure 1C,D and Figure 2D), suggesting that the APC mutation might trigger mitogen-activated signaling to promote cell survival. This data implies that 5-FU induced several pathways contributing to drug resistance by increasing genome instability and intracellular proliferation signaling.

Previous studies demonstrate that APC abolished β-catenin regulated TCF4 transcription via RAF1/MEK/ERK signaling [30,31]. Loss of APC is required for KRAS-driven CRC transformation [31] and leads to activation of the RAS-mediated cell survival pathway [32]. Moreover, Frizzleds (FZ1) ligand WNT3A, which initiates canonical WNT/β-catenin signaling, has been shown to activate ERK and stimulate cell proliferation via the RAF1/MEK/ERK pathway [33,34]. The overactivation of MEK can also be observed clinically. The ERK1/2 phosphorylation ranked as the top phosphorylation event in patients with APC truncation compared with those harboring wild-type APC in TCGA database (Figure 3). These findings demonstrate that the mitogen-activated signaling is rewired to activate and promote cell proliferation in APC-mutated cells, supporting our results of the synergic effect of MEK inhibitor and 5-FU (Figure 2D).

In the scenario of APC-independent drug resistance, we established 5-FU-resistant HCT116 cells by culturing the parental cells in the presence of an increasing dose of 5-FU over one year. To recapture the progress of developing drug resistance, we compared the gene expression profiles of 5-FU sensitive and resistant HCT116 cells. To our surprise, a *p*-value of 2.26 × 10^−54^ in the molecular function of receptor binding was remarkedly significant than any other terms in resistant cells (Table S2). Therefore, we analyzed the distribution of genes in the top five molecular functions (data not shown) to determine which is the most dominant receptor type. We found that genes were distinguished enriched in growth factor activity and cytokine receptor binding. We also noticed that growth factors, including NOV, BMP4, MDK, GDF15, VEGFC, NTF3, and LIF, were previously reported to promote cancer development, especially in cancer metastasis in various cancers, including CRC [35,36,37,38,39,40]. We suggest that cancer metastasis might develop after acquiring 5-FU resistance through the secretion of extracellular ligands modulating the microenvironment homeostasis in vivo.

The tumor microenvironment (TME) specifies critical proto-oncogenic niches to support tumorigenesis through cytokines and growth factors [41,42]. Among the growth factor activity activated in HCT116-5FUR transcriptomics analysis, IGR1R is one of the possible upstream of ROCK signaling. Concomitantly, 116-5FUR was responsive to 5-FU in the presence of the ROCK inhibitor and IGF1R inhibitor (Figure 5 and Figure S1), emphasizing the plasticity of TME in establishing drug resistance. These findings support our results that targeting TME factors improved 5-FU sensitivity in CRC cells.

## 5. Conclusions

For the APC mutation background, we applied a genetic background-based prediction to generate a list of sensitive and resistant drugs. For APC wild-type, we performed a gene expression-based method to predict drugs to reverse the 5-FU resistance phenotype. We validate that both ways provide meaningful information for combinatorial therapy and illustrate a considerable direction to implement two strategies while preparing cocktail treatment in cancer therapy. We suggest that the MEK inhibitor is the option for patients with APC truncation and ROCK inhibitor for patients with wild-type APC when 5-FU resistance develops. Taken together, we provide two independent approaches, genetic background- and gene expression-based analysis, to predict drugs that may be suitable for overcoming drug resistance, especially highlighting the importance of personalized medicine.

## Figures and Tables

**Table 1:** Predicted drugs list for setback gene expression of drug-resistant traits.

Rank	Perturbation	Target
1	RHO-kinase-inhibitor-III [Rockout]	ROCK
2	dexamethasone	steroid
3	PKCbeta-inhibitor	PKC8β
4	CYT-997	microtubule polymerization
5	AR-C133057XX	NOS2
6	NF-449	P2 receptors
7	SB-216763	GSK3 inhibitor
8	emetine	40S ribosome
9	AC-55649	RARβ2
10	FK-866	NAMPT

## Data Availability

Data can be available upon request.

## Supplementary Materials

The following are available online at https://www.mdpi.com/article/10.3390/biomedicines9080882/s1, Table S1: Functional enrichment analysis of upregulated genes in 5-FU resistant HCT116 cells, Table S2: Functional enrichment analysis of downregulated genes in 5-FU resistant HCT116 cells, Figure S1: MEK inhibitor PD-0325901 is cytotoxic to APC-mutated CRC cells, Figure S2: IGF1R inhibitor increases the response of resistant cells to 5-FU.
