# Supplementary material for: Predicting Agents That Can Overcome 5-FU Resistance in Colorectal Cancers via Pharmacogenomic Analysis
Source: Biomedicines. 2021 Jul 24;9(8):882. doi: 10.3390/biomedicines9080882 (PMC8389646; doi:10.3390/biomedicines9080882)
Supplement: Supplementary file 1 [file biomedicines-09-00882-s001.zip › biomedicines-1299049-supplementary.pdf]

Supplementary Materials

Predicting Agents that can Overcome 5-FU Resistance in Colorectal Cancers via Pharmacogenomic Analysis

Table S1. Functional enrichment analysis of upregulated genes in 5-FU resistant HCT116 cells.

**Name**	**Adj. *p*-Value (Bonferroni)**
Molecular Function	
receptor binding	2.26×10^{-54}
growth factor activity	5.52×10^{-12}
cytokine receptor binding	6.37×10^{-10}
cytokine activity	7.48×10^{-8}
hormone activity	2.89×10^{-4}
growth factor receptor binding	8.49×10^{-3}
chemoattractant activity	8.51×10^{-3}
tumor necrosis factor receptor binding	3.23×10^{-2}
protein complex binding	4.95×10^{-2}
glycosaminoglycan binding	6.58×10^{-2}
Biological Process	
cell migration	2.50×10^{-11}
localization of cell	1.47×10^{-10}
cell motility	1.47×10^{-10}
regulation of cell migration	1.41×10^{-9}
regulation of cell motility	3.34×10^{-9}
regulation of locomotion	1.48×10^{-8}
regulation of cellular component movement	1.96×10^{-8}
locomotion	2.91×10^{-8}
positive regulation of cell migration	8.60×10^{-8}
positive regulation of cell motility	1.07×10^{-7}
Cellular Component	
extracellular space from receptor binding	4.39×10^{-11}

Table S2. Functional enrichment analysis of downregulated genes in 5-FU resistant HCT116 cells.

**Name**	**Adj. *p*-value (Bonferroni)**
Molecular Function	
RNA binding	2.80×10^{-5}
poly(A) RNA binding	7.56×10^{-5}
Biological Process	
ncRNA processing	6.45×10^{-9}
ncRNA metabolic process	2.39×10^{-7}
rRNA metabolic process	1.84×10^{-6}
ribonucleoprotein complex biogenesis	2.87×10^{-6}
ribosome biogenesis	3.43×10^{-6}
rRNA processing	7.39×10^{-6}
RNA processing	1.28×10^{-3}
RNA modification	4.81×10^{-2}
Cellular Component	
nucleolus	2.51×10^{-7}

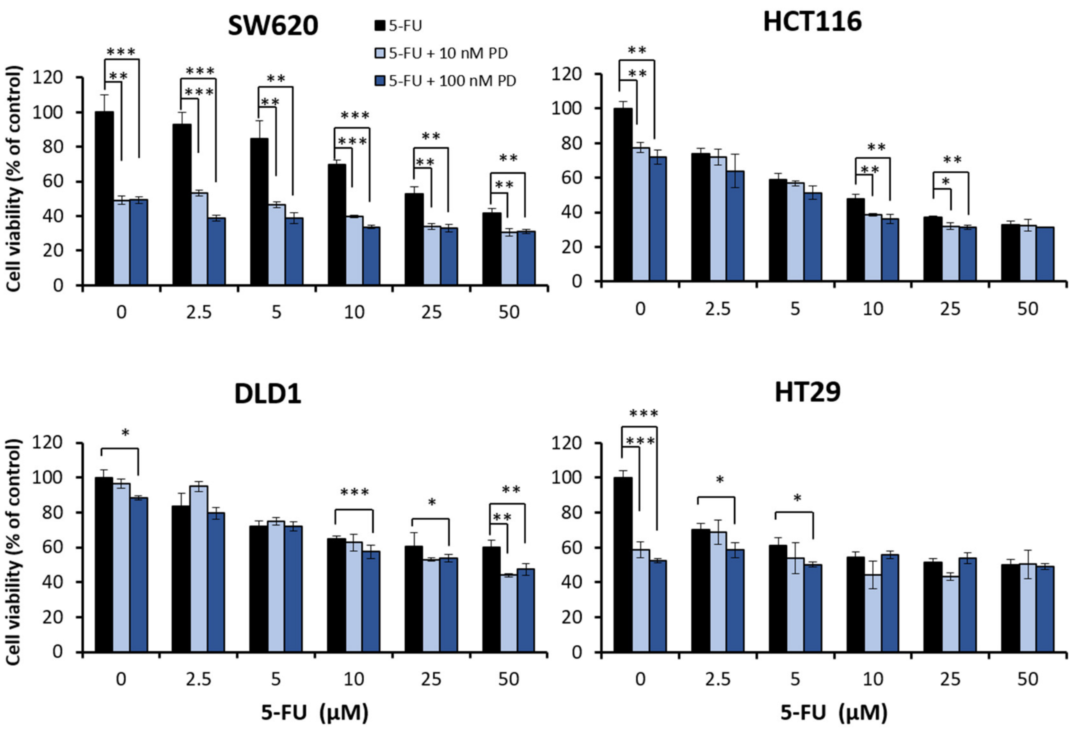

Figure S1. MEK inhibitor PD-0325901 is cytotoxic to APC mutated CRC cells. Cells were treated with 5-FU alone at indicated concentrations (black bars) or treated with 5-FU in the combination of MEK inhibitor PD-0325901 at the concentration of 10 nM, and 100 nM represent in light blue bars and dark blue bars, respectively. *, $p < 0.05$; **, $p < 0.01$; ***, $p < 0.001$; in compared with 5-FU alone treated cell viability.

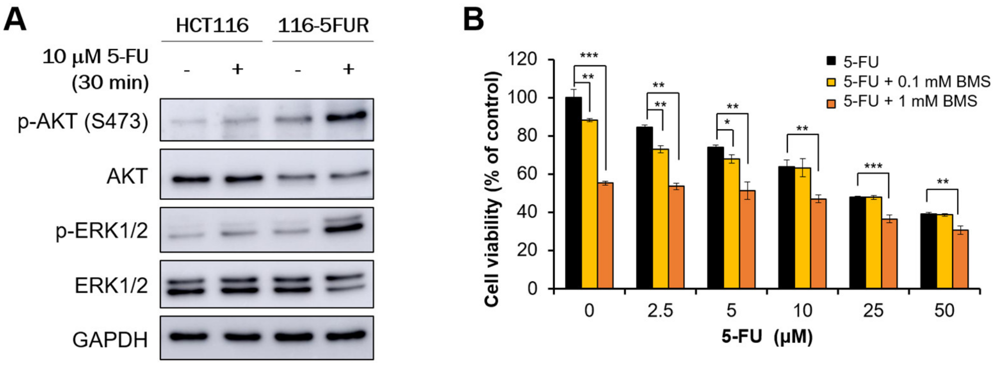

Figure S2. IGF1R inhibitor increases the response of resistant cells to 5-FU. (**A**) Cells were treated with 5-FU for 30 minutes and subjected to examine the level of IGF1R downstream signaling, AKT and ERK phosphorylation. GAPDH was the loading control. (**B**) 116-5FUR cells were treated with BMS-754807 (BMS) and 5-FU at indicated concentrations. Cell viability was measured and normalized to DMSO treated cells. Chrome yellow and orange bars show different doses of BMS. *, $p < 0.05$; **, $p < 0.01$; *** $p < 0.001$; in compared with 5-FU alone treated cell viability.
